# Evaluate enamel surface staining due to iron supplements on primary teeth - An *in vitro* study

**DOI:** 10.6026/973206300212980

**Published:** 2025-09-30

**Authors:** Anish Ashok Gupta, Rohit Sharma, Dimple Kishan Tirale, Ramakrishna Surada, Udita Samanta, Ananya Bhargava, Miral Mehta

**Affiliations:** 1 Department of Oral Pathology & Microbiology, People's Dental Academy, People's University, Bhopal, Madhya Pradesh, India; 2 Department of Dentistry, Amaltas Institute of Medical Sciences, Dewas, Madhya Pradesh, India; 3 Department of Pediatric & Preventive Dentistry, Rungta College of Dental Sciences & Research, Rungta Knowledge City, Kurud Road, Kohka, Bhilai, Chhattisgarh, India; 4 Department of Conservative Dentistry and Endodontics, Gitam Dental College and Hospital, Visakhapatnam, Andhra Pradesh, India; 5 Department of Pediatrics and Preventive Dentistry, Divya Jyoti College of dental science and Research, Modinagar, Ghaziabad, Uttar Pradesh, India; 6 Department of Dentistry, Ruxmaniben deepchand gardi medical college, Ujjain, Madhya Pradesh, India; 7 Department of Pediatric and Preventive Dentistry, Karnavati School of Dentistry, Karnavati University, Gandhinagar, Gujarat, India

**Keywords:** Iron supplements, enamel staining, primary teeth, ferrous sulfate, iron polymaltose complex, *in vitro* study

## Abstract

Iron deficiency anemia in children is commonly managed with oral iron supplements, but liquid formulations are often associated with undesirable tooth staining. This *in vitro* study evaluated enamel discoloration on primary teeth after exposure to ferrous sulfate and iron polymaltose complex. Sixty extracted primary anterior teeth were divided into three groups and immersed daily for 21 days; with color change measured using a spectrophotometer and stereomicroscopy. Ferrous sulfate caused the highest degree of staining (ΔE = 12.6), followed by iron polymaltose (ΔE = 6.3), while controls showed minimal change (ΔE = 1.2). Ferrous sulfate was associated with significantly greater enamel staining, highlighting the need to prefer low-staining formulations to improve pediatric compliance and aesthetics.

## Background:

Iron deficiency anemia (IDA) is a prevalent nutritional disorder among children worldwide and remains a significant public health issue, particularly in developing countries [1]. Oral iron supplementation is the most common and effective treatment for managing IDA in pediatric populations due to its accessibility and affordability [2]. However, despite its clinical benefits, the use of oral iron, especially in liquid formulations, has been associated with adverse effects such as gastrointestinal discomfort and dental staining [3]. Extrinsic discoloration of the enamel is a widely reported side effect of iron supplements, primarily due to the precipitation of iron compounds on the tooth surface in the oral environment [4]. This staining, though non-cariogenic may result in parental concern, aesthetic dissatisfaction, and reduced compliance with iron therapy, especially in younger children [5]. Among various iron preparations available, ferrous salts (such as ferrous sulfate) and iron polymaltose complexes differ significantly in terms of their chemical composition, absorption, and potential to cause dental staining [6]. The enamel of primary teeth is more porous and less mineralized than that of permanent teeth, making it more susceptible to staining and surface alterations due to exposure to external agents like iron [7]. Although previous studies have reported the discoloration potential of iron supplements, most of them are either observational or lack direct comparative data between different formulations on primary teeth [8, 9]. Thus, there is a growing need for controlled *in vitro* studies to quantify and compare the staining potential of commonly used iron preparations. This study aims to evaluate and compare the extent of enamel surface staining caused by two different oral iron formulations-ferrous sulfate and iron polymaltose complex-on extracted human primary teeth under standardized laboratory conditions. The findings are expected to provide evidence-based recommendations for pediatric practitioners regarding the aesthetic safety of iron therapy in children [10]. Iron-induced tooth discoloration primarily results from the interaction between iron ions and dietary chromogens or oral biofilm components, which leads to the deposition of dark-colored compounds on the tooth surface [4]. The acidic nature of iron syrups further enhances enamel surface roughness and promotes stain adherence, especially in the cervical regions and interproximal surfaces of anterior teeth, which are more visible and aesthetically significant in children [5]. Such extrinsic stains are difficulties to remove with regular brushing and may require professional cleaning, thereby increasing the burden on pediatric dental care [6]. The choice of iron formulation plays a pivotal role in determining the extent of dental staining. Ferrous sulfate is known for its high bioavailability and rapid absorption, but it is also associated with a greater propensity for causing visible tooth discoloration [7]. On the other hand, iron polymaltose complex, a non-ionic formulation, exhibits better taste tolerance and reduced gastrointestinal side effects and is often claimed to have a lower risk of staining due to its molecular stability and slower release of free iron ions [8]. However, the staining potential of these formulations on the primary dentition has not been adequately compared in controlled laboratory settings. Given that the enamel of primary teeth is thinner and less mineralized compared to permanent teeth, it is more vulnerable to extrinsic staining and surface alterations due to acidic or chromogenic substances [9]. Studies have shown that pediatric patients on long-term iron therapy may develop noticeable enamel stains, potentially impacting their self-esteem and social interactions, especially in school-aged children [1]. Hence, understanding the relative staining potential of various iron supplements is essential not only for clinical decision-making but also for improving therapeutic compliance and aesthetic outcomes. *in vitro* studies provide a standardized approach to simulate oral conditions and compare the effects of different agents on tooth surfaces. They eliminate intraoral confounding factors such as saliva composition, diet, and oral hygiene practices, thereby offering more reproducible and focused results. Therefore, it is of interest to report the comparative staining effects of ferrous sulfate and iron polymaltose complex on primary teeth in an *in vitro* setting.

## Materials and Methods:

This *in vitro* experimental study was conducted to evaluate and compare the enamel staining potential of two commonly used pediatric iron supplements on extracted primary teeth.

## Sample selection:

A total of 60 sound, caries-free, extracted human primary anterior teeth were collected from pediatric dental clinics. Teeth with developmental anomalies, restorations, hypoplastic defects, or intrinsic stains were excluded. All teeth were cleaned with an ultrasonic scaler to remove soft tissue remnants and stored in distilled water at room temperature until further use.

## Grouping and preparation:

The selected teeth were randomly divided into three groups (n = 20 per group) based on the immersion solution:

[1] Group A: Ferrous sulfate solution

[2] Group B: Iron polymaltose complex solution

[3] Group C: Control group (distilled water)

The iron solutions were prepared using commercially available pediatric formulations diluted to simulate salivary dilution during clinical use.

## Immersion protocol:

Each tooth was individually immersed in its respective solution for 5 minutes daily over a period of 21 consecutive days. Between immersions, teeth were rinsed with distilled water and stored in artificial saliva at 37°C to simulate oral conditions.

## Color assessment:

Baseline color values (L*, a*, b*) of each tooth were recorded using a digital spectrophotometer (VITA Easyshade®). After 21 days, the final color measurements were taken. The total color change (ΔE) was calculated using the formula: (see PDF)

## Surface examination:

In addition to spectrophotometric evaluation, stereomicroscopic imaging (40x magnification) was performed to qualitatively assess surface discoloration patterns and intensity.

## Statistical analysis:

Data were compiled and analyzed using SPSS software (version 25.0). Mean ΔE values were compared across the three groups using one-way ANOVA followed by post hoc Tukey's test. A p-value < 0.05 was considered statistically significant.

## Results:

A total of 60 primary anterior teeth were analyzed to assess the degree of enamel discoloration after exposure to different iron supplements over a 21-day period. Spectrophotometric evaluation showed a noticeable color change in Groups A and B, with Group A (Ferrous sulfate) showing the highest level of staining. The mean ΔE (color change) values are presented in Table 1 (see PDF). Group A exhibited the highest mean ΔE value of 12.58 ± 1.82, followed by Group B with 6.35 ± 1.41, whereas Group C (control group) showed minimal color change, 1.19 ± 0.52. Statistical analysis using one-way ANOVA revealed a significant difference (p < 0.001) in color change among the three groups. Post hoc Tukey's test further confirmed that each group differed significantly from the others (p < 0.05) as shown in Table 2 (see PDF). Visual assessment under stereomicroscopy revealed dark brown surface staining in Group A samples, mild yellowish discoloration in Group B, and no visible change in Group C. A qualitative summary of microscopic observations is detailed in Table 3 (see PDF). The correlation between spectrophotometric ΔE values and visual scoring was strong and statistically significant (r = 0.89, p < 0.001), confirming the reliability of both methods as presented in Table 4 (see PDF). These results clearly demonstrate that ferrous sulfate has a significantly higher staining potential compared to iron polymaltose complex and control.

## Discussion:

Iron supplementation is critical in managing iron deficiency anemia, particularly in pediatric populations where growth demands and nutritional deficiencies are common [1]. However, one of the main drawbacks of oral iron therapy, especially in liquid forms, is its potential to cause undesirable extrinsic tooth staining. The present *in vitro* study confirmed that ferrous sulfate caused significantly greater enamel discoloration on primary teeth than iron polymaltose complex, aligning with earlier clinical and laboratory-based observations [2, 3]. The highest mean ΔE value in this study was observed in the ferrous sulfate group, suggesting that this formulation has a higher tendency to release free iron ions that interact with oral chromogens or oxidize into dark-colored ferric compounds upon exposure to saliva or air [4]. This reaction contributes to the deposition of iron on the enamel surface, leading to pronounced staining. Iron polymaltose, being a non-ionic and more stable complex, demonstrated significantly less discoloration, which may be attributed to its lower free iron ion availability and reduced reactivity [5]. The thin, porous, and less mineralized enamel structure in primary teeth likely increases their susceptibility to staining, especially when exposed repeatedly to staining agents like iron formulations [6]. Previous studies have also shown that enamel porosity enhances the adsorption of iron particles and other chromogens; exacerbating the discoloration process in pediatric patients [7]. So also pain control seems to be the most important this during this process [8]. Iron supplements, though essential for managing childhood anemia, often cause noticeable staining on primary teeth. Protective enamel coatings, such as varnishes and resin-based surface sealants, can help reduce this discoloration, offering a preventive approach to maintain aesthetics in pediatric dentistry [9]. Enamel surface coatings significantly reduce the staining effect of iron-containing supplements teeth.

## Conclusion:

Ferrous sulfate causes significantly higher enamel discoloration in primary teeth compared to iron polymaltose complex. Thus, iron polymaltose may be a preferable option in pediatric patients to minimize aesthetic concerns. Hence, clinicians should consider the staining potential of iron supplements when prescribing long-term therapy.
